# Directed Fiber Outgrowth from Transplanted Embryonic Cortex-Derived Neurospheres in the Adult Mouse Brain

**DOI:** 10.1155/2009/852492

**Published:** 2010-02-14

**Authors:** Vesna Radojevic, Josef P. Kapfhammer

**Affiliations:** ^1^HNO Klinik, ZLF 411, University of Basel, Hebelstr. 20, 4031 Basel, Switzerland; ^2^Department of Biomedicine, Anatomical Institute, University of Basel, Pestalozzistr. 20, 4056 Basel, Switzerland

## Abstract

Neural transplantation has emerged as an attractive strategy for the replacement of neurons that have been lost in the central nervous system. Multipotent neural progenitor cells are potentially useful as donor cells to repopulate the degenerated regions. One important aspect of a transplantation strategy is whether transplanted cells are capable of fiber outgrowth with the aim of rebuilding axonal connections within the host brain. To address this issue, we expanded neuronal progenitor from the cortex of embryonic day 15 ubiquitously green fluorescent protein-expressing transgenic mice as neurospheres in vitro and grafted them into the entorhinal cortex of 8-week-old mice immediately after a perforant pathway lesion. After transplantation into a host brain with a lesion of the entorhino-hippocampal projection, the neurosphere-derived cells extended long fiber projections directed towards the dentate gyrus. Our results indicate that transplantation of neurosphere-derived cells might be a promising strategy to replace lost or damaged axonal projections.

## 1. Introduction

A variety of diseases and insults to the nervous system will eventually result in the loss of functional connections between neurons (axonal lesion) or to the complete loss of neurons (neurodegenerative or vascular lesion). In both conditions, the affected system will become increasingly dysfunctional resulting in permanent functional deficits. This happens, because a disease- or injury related loss of neurons or axonal connections in the adult and aged brain is usually not followed by adequate self-repair or appropriate reorganization of spared axonal connections [1, 2]. One important factor for functional improvement is to achieve axonal regeneration of lesioned axons in order to restore neural circuits [3, 4].

The grafting of fetal neural cells committed to specific neuronal phenotypes into appropriate sites in the damaged young or aged brain has been found to be useful for both facilitating the repair of disrupted circuits and preventing the formation of inappropriate synaptic reorganization [5, 6]. Recently, it was shown that after transplantation of fetal motor cortex into adult hosts, long fiber projections could be specifically rebuilt [7]. The neuronal progenitor cells also seem to support self-repair of the adult brain after injury or disease [8–11]. Pioneer studies for the treatment of Parkinson's disease have shown that the transplantation of embryonic neural precursor cells has indeed the potential of achieving a good functional restoration for some patients [12], but major ethical and practical problems limit the use of human embryonic neural grafts [8]. One important alternative is the use of cultured neuronal stem cells. In previous studies, grafting of neurosphere-derived stem cells was shown to be feasible in several models of neuronal injury. The grafted cells were shown to be able to survive for long periods of time and differentiate into neurons and glial cells [13–16]. In addition, functional improvements have been reported after transplantation of neurosphere-derived cells in some studies [17, 18]. In a previous in vitro study using organotypic slice-culture of the entorhino-hippocampal formation, we have shown that transplants of embryonic cerebral cortex were able to form specific projections to the dentate gyrus of the hippocampus [19]. We have now extended these studies and explored the potential of transplanted embryonic cortex-derived neurospheres to form fiber projections in vivo. Our results show that neurosphere-derived cells transplanted after a lesion of the perforant path projection developed a substantial fiber projection which was specifically directed towards the host hippocampus.

## 2. Materials and Methods

All procedures involving animal care were conducted in conformity with the European Communities Council Directive of 24 November 1986 (86/609/EEC) and were reviewed and permitted by Swiss authorities.

### 2.1. Preparation and Maintenance of Neurospheres

Neural stem cells were isolated from E15 embryos of timed pregnant mice expressing a Tau-GFP fusion protein [20] which were backcrossed to the C6BF1 mice used as hosts. The fetal mouse brain was removed and placed in preparation medium (PM) consisting of MEM with 2 mM glutamax at pH 7.3. The embryonic cortex from both hemispheres was dissected. The tissue was cut into 2 mm cubes and transferred to a sterile 15 mL tube. Cortical tissue from a litter was pooled and transferred to fresh PM. Dissected tissue pieces were rinsed twice in PM and trypsinized for 15–20 minutes at 37°C. Trypsinization was stopped by addition of horse serum (one-quarter volume) and DNase (0.01%). The cells were then centrifuged in a bench centrifuge for 5 minutes at 600 g at room temperature. The pellet was re-suspended and 100,000 cells/mL were cultured in serum-free complete growth medium 1 (GM1) in the presence of epidermal growth factor and basic fibroblast growth factor (both 10 ng/mL) [21]. GM1 consisted of neurobasal medium (Gibco), D-glucose (25 mM), L-glutamin (1 mM), B-27 supplement (Gibco), 0.25 mM Glutamax (Gibco), penicillin G (50 U/mL), and streptomycin/ampicillin (50 *μ*g/mL). After approximately 7 days of culture, cells had grown to free-floating neurospheres. For passaging, spheres were dissociated mechanically and were resuspended in the same medium at a density of 50.000 cells/mL. Trypan blue exclusion indicated that this preparation consisted of 87–98% viable cells. 

The differentiation potential of neurospheres-derived cells was investigated by plating them onto poly-L-lysine coated culture dishes for 7 days. Culture dishes were coated with 50–100 *μ*L of 10 *μ*g/mL poly-D-lysine, and pretreated with 10% heat inactivated FCS for 2 hours. The cells were seeded at a density of   0.5–1.0 × 10^6^ cells/cm^2^ and incubated in differentiation medium (GM1 containing BDNF, Cell Concepts, 20 ng/mL) at 37°C. The cultures were fed every 4–6 days. 

### 2.2. Surgical Procedures

Young adult (16–20-week-old) female C6BF1 mice were anesthetized with an intraperitoneal (IP) dose of Ketamine (Intervet, Zurich, 0,08 g/kg) and Climasol (Graeub, Berne, 5 mg/kg) after a light sedation with Temgesic (Essex Chemie – Lucerne, 0,1 mg/kg) and Atropine (Sintetica S.A., Mendrisio, 0,05 mg/kg). During anesthesia, animals were given a subcutaneous (SC) dose of Metacam (Böhringer Ingelheim, 1 mg/kg) as a postsurgical anti-inflammatory agent. The mice were placed in a stereotaxic apparatus and a hole was drilled in the skull. The wire-knife was fitted onto the stereotaxic apparatus at the coordinates AP + 0.25 mm, L 0.05 mm and V - 0.45 mm above ear zero plane according to the atlas of Franklin and Paxinos [22]. The wire-knife was then inserted at a 110 lateral angle. Four millimeters ventral to the Dura, the wire was unfolded and the perforant path was sectioned by retracting the knife 3.2 mm. For the cell injection, a Hamilton needle was inserted 0.6 mm posterior and 1.9 mm lateral from the bregma. Two microliters of neurosphere suspension with approximately 100,000–200,000 cells were injected using a Hamilton syringe into the lateral entorhinal cortex. After transplantation, the wound was closed, a salt solution was injected subcutaneously, and mice were allowed to recover in a heated cage and returned to the animal facilities when fully awake.

### 2.3. Histology and Immunohistochemistry

After 21 days, mice were killed with an overdose of sodium pentobarbital (100 mg/kg) and transcardially perfused with 4% paraformaldehyde (pH 7.4, at 4°C). The brain was then removed from the skull and postfixed over night. For histological evaluation, coronal sections of 30 *μ*m thicknesses were cut on a vibratome and mounted on Superfrost plus slides (Menzel, Germany). Neurospheres after 7 and 10 days of culture attached to PLL-coated cover slips were fixed in 4% paraformaldehyde solution for 15 minutes, washed twice, and kept in 0,01 M phosphate-buffered saline (PBS) at 4°C for further processing. Vibratome sections and neurosphere cultures were incubated for 1 hour at room temperature in blocking solution containing PBST and 3% normal goat serum followed by the first antibody over night at 4°C. The following antibodies were used: Rabbit polyclonal antibody against glial fibrillary acidic protein GFAP (DAKO), mouse-monoclonal antibody SMI-31 against phosphorylated neurofilaments (Sternberger Incorporated), mouse-monoclonal antibody against NeuN and *β*-III-tubulin (Chemicon), and rabbit polyclonal antibody against NG2 (Millipore). After 3 washes in PBS, the sections or cells were incubated for 1 hour at room temperature with the appropriate secondary antibodies (1 : 250, Alexa conjugated, Molecular Probes) diluted in PBST with 1% NGS for 2 hours at room temperature.

## 3. Results and Discussion

### 3.1. Neurosphere-Derived Cells Differentiated into Astrocytes and Neurons In Vitro

**Figure 1:**
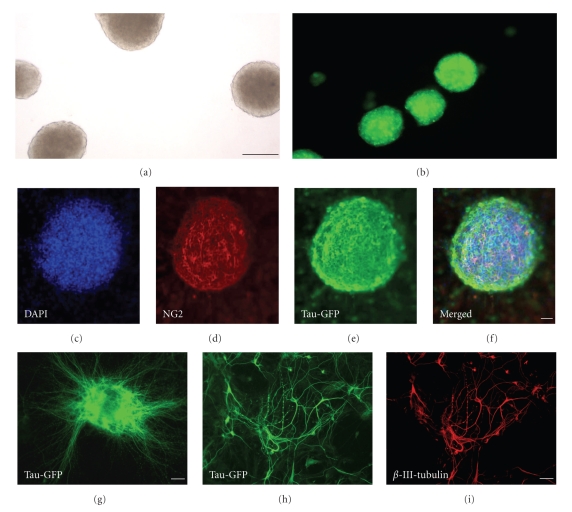
Analysis of neurosphere growth and differentiation in vitro. (a) Low power view (bright field) of a neurosphere culture. Many cortex-derived neurospheres had developed after 7 days in vitro. Scale bar = 50 *μ*m. (b) Neurospheres expressing a Tau-GFP fusion protein from the cortex of Tau-GFP transgenic mice. (c) DAPI staining of a neurosphere culture. (d) Within the cultured neurospheres, NG2 positive cells (red) were typically located in the center of the rosettes. (e) Tau-GFP expression was present throughout the neurospheres. (f) Merged image between DAPI, Tau-GFP, and NG2 showing the central location of NG2 positive cells within the neurosphere. Scale bar = 20 *μ*m. (g) One week after transfer to poly-D-lysine-coated dishes, Tau-GFP-positive neurosphere-derived cells had attached and extended processes. Scale bar = 20 *μ*m. (h) Network of cellular processes from neurosphere-derived cells as seen with Tau-GFP expression. (i) Staining with the neuronal marker *β*-III-tubulin (H) confirms the neuronal differentiation of many neurosphere-derived cells. Scale bar = 50 *μ*m.

Neural progenitor cells isolated from GFP-expressing embryonic cortex proliferated in response to growth factors in the culture medium and formed neurospheres. After seven days in vitro, these cells formed clusters measuring about 50–90 *μ*m in diameter (Figures 1(a) and 1(b)). Many of the neurospheres contained glial precursor cells visualised by GFAP (data not shown) or by staining with NG2 (Figures 1(c) and 1(d)) which were typically located in the more central parts of the neurospheres. Tau-GFP expression as expected was ubiquitously present throughout the neurospheres (Figures 1(e) and 1(f)). The ability of neurosphere cells for robust neuronal differentiation was assessed through direct culturing of neurospheres in substrate (poly-L-lysine) coated Petri plates containing differentiation medium (GM1 plus BDNF). One week after plating, many cells had differentiated into a typical neuronal morphology with an axon, growth cones, and a beginning dendritic arborization (Figures 1(g)–1(i)). Immunostaining for markers of neuronal (*β*-III-tubulin), astrocyte (GFAP) (data not shown), and oligodendrocyte antigens (NG2) (data not shown) clearly showed the presence of all three CNS cells types among the differentiated cells derived from neurospheres. Under our standard condition of differentiation, we obtained 50–70% of neuronal lineage (*β*-III-tubulin), 15–20% astrocytic lineage (GFAP), and 10–20% oligodendrocyte lineage (oligodendrocyte progenitor marker NG2) as determined by manual counting of dissociated immunolabeled cells.

### 3.2. Transplanted Neurosphere-Derived Cells Differentiated into Neurons and Formed Fiber Projections Directed towards the Lesioned Entorhino-Hippocampal Formation

**Figure 2:**
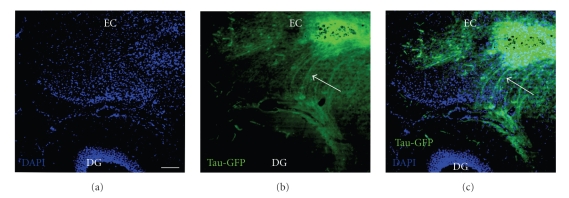
A neurosphere transplant after 3 weeks survival. The transplant is located in the entorhinal cortex (EC), and a strong fiber bundle directed towards the dentate gyrus has developed. GFP positive labeled fibers mostly stop in the area of the hippocampal fissure. (a) DAPI staining. Scale bar = 100 *μ*m. (b) GFP labeled neurosphere transplant with fiber bundle. (c) Combined image.

**Figure 3:**
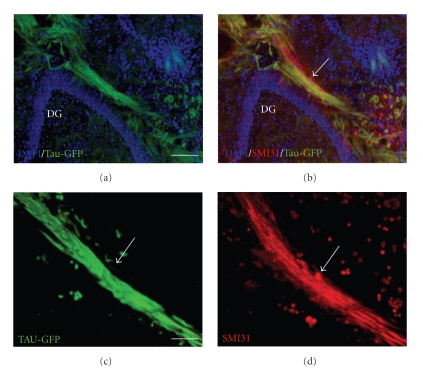
Projection from neurosphere-derived cells towards the lesioned entorhino-hippocampal formation. (a) A strong transplant-derived GFP-positive fiber bundle extends towards the dentate gyrus of the hippocampus in the area of the outer molecular layer. Scale bar = 50 *μ*m. (b) Combined image of GFP and SMI-31 confirms that the GFP-expressing fiber bundle (arrow) contains SMI-31-positive axons. (c) High magnification of GFP-positive fibers (arrow) from the transplant in the area of the hippocampal fissure and the outer molecular layer of the dentate gyrus. Scale bar = 100 *μ*m. (d) SMI-31 staining of GFP-positive fibers (arrow) from the transplant.

**Figure 4:**
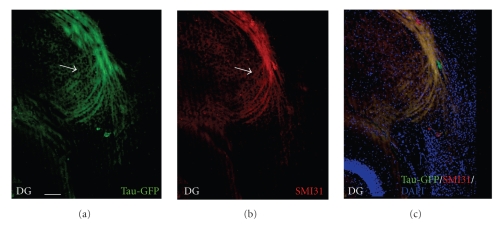
Another projection from neurosphere-derived cells towards the lesioned entorhino-hippocampal formation. The location of the dentate gyrus (DG) is indicated in a–c. (a)–(c) A GFP-positive fiber bundle in the area of hippocampal fissure with coexpression of SMI-31. Fibers end in the region of the hippocampal fissure. (a) Tau-GFP. Scale bar = 50 *μ*m. (b) SMI-31 staining. (c) Combined image.

In adult mice, the perforant path was lesioned with a wire knife in order to denervate the dentate gyrus. This lesion method was shown previously to result in rather complete loss of entorhinal afferents to the hippocampal formation [23]. Cultured neurospheres were injected close to the lesion site into the entorhinal cortex. Neurosphere grafts survived well when transplanted immediately after a mechanical lesion. This finding is in agreement with previous studies [24, 25]. Three weeks after the injection, many of the neurosphere-derived cells expressed the neuronal marker NeuN (data not shown). Short processes were emanating from most of the transplants in all directions. In 11 out of 20 cases, transplant-derived processes formed a fiber bundle which grew towards the denervated dentate gyrus. An example of such a fiber bundle directed towards the dentate gyrus is shown in Figures 2(a)–2(c). GFP positive labeled fibers could be seen to form a loose bundle, which took a directed path towards the dentate gyrus (Figures 2(a)–2(c)). While the GFP-expression in the transplanted cells allowed clear identification of fiber bundles, it was not sufficient to resolve individual regenerating fibers with high-power objectives. In Figure 3, a strongly GFP-positive bundle is shown. Labeling with SMI-31, a neurofilament antigen predominantly present in axons [26], suggests that this bundle contains transplant-derived axons (Figures 3(a)–3(d)). A further example of a GFP-positive fiber bundle arising from the transplant is shown in Figures 4(a)–4(c). The fibers in this bundle turned in a directed way towards the dentate gyrus (arrows in Figures 4(a)–4(c)). This bundle was also SMI-31 positive (Figure 4(b)). In the examples shown, the fibers formed a bundle which was specifically directed towards the host dentate gyrus. While the majority of these axons reached the area of the hippocampal fissure, they did not branch extensively in the outer molecular layer of the dentate gyrus (Figures 4(a)–4(c)) but rather stopped in the area of the hippocampal fissure. Preferential growth towards the dentate gyrus was observed in all cases which showed fiber outgrowth, that is, in 11 out of 20 transplants.

An important aspect of neurosphere transplantation is to examine whether grafted neurospheres have a propensity for tumor formation in the injured aged brain [27]. In order to exclude tumor formation during the survival period, we examined the expression of von Willebrand factor in 5 grafts after 3 weeks. These immunostainings yielded consistently negative results (data not shown). The negative immunostaining with von Willebrand factor which has epigenetic effects on angiogenesis in cancer shows that our graft at 3 weeks did not give rise to tumor tissue [27, 28]. It does not rule out a long-term tumorigenic potential of the transplanted cells.

## 4. Conclusions

In this study, we examined the potential of immature neurosphere-derived neural progenitor cells to form fiber projections within the adult mouse brain. In some cases, transplanted cells extended a fiber projection which appeared to be selectively and specifically directed towards the denervated dentate gyrus. Most of these fibers reached the hippocampal fissure but little invasion of the outer molecular layer of the dentate gyrus was observed. Our results show that immature neural cells which were propagated in culture have the potential to extend a fiber projection directed towards a denervated target area.

### 4.1. Transplanted Neurospheres Differentiate into Neurons and Are a Source of Immature Neuronal Precursor Cells for Transplantation

In recent years neurosphere cultures [29, 30] have emerged as an attractive source of immature neuronal cells for neural transplantation. The use of cultured neurospheres reduces the ethical and practical problems associated with transplantation of fresh human embryonic material [31]. In this study, we have used neurospheres derived from the cerebral cortex of E15 mouse embryos. As expected, the neurospheres yielded a mixed population of neuronal and glial cells as has been described previously [30]. For the purpose of transplantation, this mixed differentiation of cells could be of advantage because survival and differentiation of transplanted neurons might be stimulated by glial cells from the transplant. 

We have analyzed the differentiation of neurosphere-derived cells in vitro. Our finding that the SMI-31 positive cells also show morphological characteristics of neurons confirms the good potential of these cells for neuronal differentiation. This neuronal phenotype is compatible with the finding that neurospheres-derived cells show electrophysiological properties of immature neurons [32]. Neurospheres cultured from embryonic cerebral cortex thus appear to be a promising source for neuronal precursor cells, suitable for transplantation studies.

### 4.2. Substantial Fiber Outgrowth from Transplanted Neuronal Precursor Cells in the Adult Brain

In this study, we have observed substantial fiber outgrowth from the transplanted neurosphere-derived neuronal precursor cells which was directed towards and reached the appropriate target region. In some previous studies, process outgrowth was reported to be rather sparse after transplantation of grafted neurons into the adult brain. Using embryonic dopaminergic precursor cells good fiber outgrowth could only be achieved after transplantation into young postnatal hosts, but not in adult hosts [33]. This is in an agreement with a general decline of axonal outgrowth and regeneration with increasing age [34]. Good axonal outgrowth and the formation of long range functional connections were reported after transplantation of a neural progenitor cell line into the neonatal brain [35]. There are only few reports of good fiber outgrowth after transplantation into adult hosts. When fetal hippocampal cells were injected into the CA3 region of kainic acid-lesioned adult rats, projections from the transplant reached the CA1 region and the dentate gyrus [6]. In our experiments, after a survival time of three weeks, in several cases a fiber bundle emanated from the transplanted cells and extended towards the dentate gyrus. This is in agreement with a recent study [7] demonstrating extensive fiber outgrowth after transplantation of embryonic motor cortex in the adult mouse brain. Our observation that many transplant-derived axons had stopped at the hippocampal fissure may be due to inhibitory cues, which prevented the axons from crossing this border. A failure of entorhinal axons to cross the hippocampal fissure was reported in rodents with mutations in the *reelin* gene, the SRK rat [36], and the reeler mouse [37].

### 4.3. Transplanted Neurosphere-Derived Neuronal Precursors Cells May Have the Potential for Axonal Repair

Recently, neurosphere grafting has been perceived as a potential therapeutic approach for alleviating age-related neurodegenerative disorders [38]. In a previous in vitro study, we could show that embryonic cortex-derived axons formed a specific projection to the dentate gyrus in entorhino-hippocampal slice cultures, and that this specific projection formed irrespective of the regional origin of the embryonic cortex [19]. Encouraged by this finding, we have now used even more immature neurosphere-derived neuronal precursor cells and could show that these cells after transplantation into the lesioned brain are indeed capable of generating fiber outgrowth which projected over a considerable distance towards the appropriate denervated target area. The results of this study suggest that transplanted neurospheres can form fiber bundles in the adult brain, and that neurosphere cultures might be a promising and convenient source of neuronal precursor cells for neural transplantation.
